# Facile synthesis of hybrid CNTs/NiCo_2_S_4_ composite for high performance supercapacitors

**DOI:** 10.1038/srep29788

**Published:** 2016-07-11

**Authors:** Delong Li, Youning Gong, Chunxu Pan

**Affiliations:** 1 Shenzhen Research Institute, Wuhan University, Shenzhen 518057, China; 2 School of Physics and Technology, and MOE Key Laboratory of Artificial Micro- and Nano-structures, Wuhan University, Wuhan 430072, China; 3 Center for Electron Microscopy, Wuhan University, Wuhan 430072, China

## Abstract

In this work, a novel carbon nanotubes (CNTs)/NiCo_2_S_4_ composite for high performance supercapacitors was prepared via a simple chemical bath deposition combined with a post-anion exchange reaction. The morphologies and phase structures of the composites were characterized using scanning electron microscopy (SEM), X-ray diffraction (XRD), Raman spectroscopy (Raman), X-ray photoelectron spectroscopy (XPS) and low-temperature sorption of nitrogen (BET). The electro-chemical tests revealed that the CNT/NiCo_2_S_4_ composite exhibited high electrochemical performance, because the CNTs were used as a conductive network for the NiCo_2_S_4_ hexagonal nanoplates. Compared with pure NiCo_2_S_4_ and the mechanically mixed CNTs/NiCo_2_S_4_ composite, the CNTs/NiCo_2_S_4_ composite electrode material exhibited excellent supercapacitive performance, such as a high specific capacitance up to 1537 F/g (discharge current density of 1 A/g) and an outstanding rate capability of 78.1% retention as the discharge current density increased to 100 A/g. It is therefore expected to be a promising alternative material in the area of energy storage.

Supercapacitors have attracted considerable attentions because of their fast recharge capability, higher power density, and long cycling lifespan, all of which make them an essential power source for various energy storage applications^1^,^2^,^3^. In general, the electrode materials applied in supercapacitors can be divided into two categories based on their energy storage mechanisms: electrical double layer capacitors (EDLCs) and pseudo-capacitors (PCs)^3^. The performance of supercapacitors mainly depends on the electrochemical properties of the electrode materials. In fact, PCs usually exhibit much higher capacitances than EDLCs, because PCs electrode materials provide energy by fast and reversible redox reactions^4^.

Transition metal sulphide (or oxide) exhibits ultra-high pseudoactive capacitance among the various electrode materials^5^,^6^, and has a much higher specific capacitance due to its multiple oxidation states. However, the poor electrical conductivity and low energy density of these materials restrict their applications^7^. Therefore, it is imperative to develop high performance alternative materials for supercapacitors.

Recently, a type of ternary nickel cobalt sulphide (NiCo_2_S_4_) has been demonstrated as a promising electrode material for supercapacitors. NiCo_2_S_4_ has possess richer redox reactions than the corresponding binary nickel sulphide and cobalt sulphide^8^. Compared with NiCo_2_O_4_, NiCo_2_S_4_ shows a narrower band gap and higher conductivity, which indicate better electrochemical performance^7^,^8^. Park *et al*.^9^. determined that the replacement of oxygen (O) with sulphur (S) creates a more flexible structure because the electronegativity of Sis lower than that of oxygen (O), which prevents the disintegration of the structure by the elongation between the layers followed by the enchancement of the electron transport efficiency in the structure. These advantages make NiCo_2_S_4_ a promising electrode material for application in supercapacitors and Li-ion batteries. However, reaching its high theoretical capacitance is still a challenge because of the difficulty in controlling the microstructures, chemical compositions, shapes, morphologies, and structural instability^10^,^11^.

In general, an efficient way to overcome the above problems is to prepare a hybrid composite with highly conductive materials, such as nanoporous carbons, carbon nanotubes (CNTs), and graphene sheets^12^,^13^,^14^,^15^. These conductive additives can not only buffer the larger volume expansion and agglomeration of the active electrode materials during the charge/discharge processes, but also enhance the electrical conductivity^13^,^14^,^15^. CNTs have been considered promising materials for the fabrication of soft and conductive devices. In addition, CNTs have also been considered promising candidates for high performance electrode materials with a conductive network in the composites, because of their excellent conductivity, high specific surface area, high strength, chemical stability and low density^16^,^17^. Recently, several types of CNTs involved composites have been prepared, such as CNTs/MnO_2_^18^, CNTs/CoO^19^, and CNTs/NiCo_2_O_4_^14^,^20^,^21^.

In this study, we successfully prepared a novel hybrid CNTs/NiCo_2_S_4_ composite via a simple chemical bath deposition combined with a post-anion exchange reaction. The introduction of CNTs greatly enchance the electrochemical properties of NiCo_2_S_4_, including a large specific capacitance, good cycling stability, and excellent rate capability. In addition, this preparation strategy is also expected to obtain a type of soft and conductive CNTs involved metal sulphide composite with a controllable structure for promising materials for high performance supercapacitors.

Figure 1(a) illustrates XRD patterns of pure NiCo_2_S_4_ and the CNTs/NiCo_2_S_4_ composite. The diffraction peaks at 2 theta = 26.5°, 31.7°, 38.1°, 50.3°, and 55.1° were indexed as the crystal planes (220), (331), (400), (511), and (440) of NiCo_2_S_4_, respectively. However, the diffraction peaks of the CNTs could not be clearly identified because of their low content and small atomic number. In the Raman spectra of the CNTs/NiCo_2_S_4_ composite, as shown in Fig. 1(b), the peaks of the CNTs were clearly observed at 1353.5 1575.3 and 2704.4 cm^−1^, which are corresponding to the D, G and 2D band, respectively^20^, whereas the peaks at 516.3, and 668.7 cm^−1^ belong to the F2g and A1g modes of NiCo_2_S_4_^11^,^22^.

X-ray photoelectron spectroscopy (XPS) is an effective technique for analyzing the surface species and chemical states of elements. Figure 2 illustrates the XPS spectra of the CNTs /NiCo_2_S_4_ composite. Obviously, the C1s, S1s, Ni2p and Co2p peaks originate from the composite, and the following conclusions were obtained: 1) Fig. 2(a) shows two main peaks, which correspond to SP^2^ C = C (C1, ~284.6 eV) and C-OH (C2, ~285.6 eV) bonds, whereas the weak fitting peak at a binding energy of 288.4 eV (C3) is ascribed to a C-O bond^23^,^24^. 2) As shown in Fig. 2(b), the S1s peak can also be divided into two main peaks and one shake-up satellite peak, where the component at 163.8eV is typical of a metal-sulphur bond, whereas the component at 162.6eV is attributable to the sulphur ion in low coordination on the surface^8^. 3) By using Gaussian fitting, the Ni 2p spectrum, shown in Fig. 2(c), is fitted considering two spin-orbit doublets characteristic of Ni^2+^ and Ni^3+^ and two shakeup satellite peaks. According to the fitted data, the peaks at 854.1 eV and 872.7 eV are indexed to Ni^2+^, whereas the peaks at 855.9 eV and 875.0 eV are ascribed to Ni^3+^; 4) In the Co 2p spectrum, as shown in Fig. 2(d), two types of Co species were also observed. The fitted peaks at 781.9 eV and 796.7 eV are indexed to Co^2+^, whereas the other two peaks at 779.7 eV and 795.1 eV belong to Co^3+^. Additionally Co^3+^/Co^2+^ coexist in the composite. These results match well with the reported data of the Co 2p and Ni 2p spectra in NiCo_2_S_4_^8^,^25^. In addition, the existence of Co^3+^/Co^2+^ and Ni^3+^/Ni^2+^ cations in the CNTs/NiCo_2_S_4_ composite provides abundant active sites for energy storage.

Figure 3 shows the SEM morphologies of the CNTs/NiCo_2_S_4_ composite. Obviously, NiCo_2_S_4_ forms hexagonal nanoplates with sizes of 100–250 nm in length and approximately 50 nm in thickness (see Figure S1). The CNTs additives were uniformly dispersed among the NiCo_2_S_4_ nanoplates. The SEM morphologies of CNTs/NiCo_2_S_4_ with different mass ratio of CNTs were shown in Figure S2. The present CNTs/NiCo_2_S_4_ composite exhibited different morphologies from the reported core-shell structures^14^,^21^,^26^: i.e., CNTs in the present composite built a three-dimensional (3-D) network structure, and the NiCo_2_S_4_ hexagonal nanoplates were surrounded by the network. Therefore, this network possesses the following advantages: 1) the CNTs worked as charge carrier transport channels, making the network conductive; 2) it provided massive electro-active sites for the charge-discharge reaction; 3) it effectively prevented the agglomeration of NiCo_2_S_4_ nanoplates and ensured the full utilization of the electro-active materials; and 4) it was in favour of eliminating the volume change of the electrode materials during the charge-discharge reaction. In summary, this network played an important role in improving the conductivity and stability of the electrode materials.

The BET test was performed to evaluate the specific surface area and pore-size distribution of the CNTs/NiCo_2_S_4_ composite and pure NiCo_2_S_4_, as shown in Fig. 4. From the profiles, the specific surface areas of the CNTs/NiCo_2_S_4_ and NiCo_2_S_4_ were calculated to be 108.03 m^2^/g and 118.49 m^2^/g, respectively. There was no obvious difference between the materials, which reveals that the surface area was not a key factor in determinging the electrochemical properties. The structures were further characterized using Barrette Joynere Halenda (BJH) pore size distribution data, as shown in the inset of Fig. 4. The pore distributions were relatively narrow and mainly centred in the range of 2 to 5 nm for both samples, which indicates a high specific surface area, and provided rich electro-active sites and short diffusion paths for charge transports. These features are believed to be extremely beneficial for application in supercapacitors.

In general, the electrochemical properties of electrode materials can be determined using various techniques, such as cyclic voltammetry (CV), galvanostatic charge/discharge (GCD) curves and electrochemical impedance spectroscopy (EIS) in a three-electrode system. To demonstrate the improvement that the CNTs network imparted on the electrochemical properties of the composites, pure NiCo_2_S_4_ and mechanically mixed CNTs/NiCo_2_S_4_ were also measured for comparison. The electrochemical properties of the CNTs/NiCo_2_S_4_ with different mass ratio of CNTs were tested, as shown in figure S3.

Figure 5(a) illustrates the CV curves of the CNTs/NiCo_2_S_4_ composite at various scan rates of 5, 10, 20, 30, 40 and 50 mV/s. A pair of redox current peaks was obtained in all CV curves and a typical pseudocapacitance appeared in all the CV curves, which was quite different from the electric double-layer capacitance with a rectangular CV shape^27^. These redox peaks mainly originate from the Faradaic redox reactions related to Co^2+^/Co^3+^ and Ni^2+^/Ni^3+^ redox couples, which were probably mediated by the OH· ions in the alkaline electrolyte^27^,^28^. The reversible redox reaction can be expressed by the following reaction equations^7^,^29^:


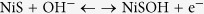



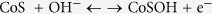



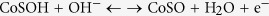


Figure 5(b) shows the CV curves of the CNTs/NiCo_2_S_4_, composite, pure NiCo_2_S_4_ and the mechanical mixed CNTs/NiCo_2_S_4_ composite at a scan rate of 5 mV/s The integrated area covered by the CV curves of the CNTs/NiCo_2_S_4_, composite was much larger than that of the other two samples, which indicates the outstanding electrochemical performance of the composite.

To evaluate the application potential of the CNTs/NiCo_2_S_4_ composite as an electrode for supercapacitors, GCD measurements were performed at various current densities from 1 A/g to 100 A/g, as shown in Fig. 5(c). The corresponding specific capacitances of the CNTs/NiCo_2_S_4_ electrode was calculated based on the charge/discharge curves, the results of which are plotted in Fig. 5(d). For comparison, the specific capacitances of pure NiCo_2_S_4_ and the mechanically mixed CNTs/NiCo_2_S_4_ composite are also plotted in Fig. 5(d). The results reveal that the specific capacitance decrease as the current density increases, which occurs beacause the redox reaction between Ni/Co cations and OH anions is a diffusion-controlled process through the electrode grain boundaries^30^.

When the discharge current densities were 1 A/g and 100 A/g, the specific capacitances of the CNTs/NiCo_2_S_4_ composite were 1537 F/g and 1200 F/g, respectively. Over this range, the specific capacitance only decreased to 78.1% of its initial value, which showed much higher values than that of the mechanically mixed CNTs/NiCo_2_S_4_ composite (55.8%) or pure NiCo_2_S_4_ (47.6%). This result is attributable to the relatively poor conductivity of pure NiCo_2_S_4_ and loose contact between CNTs and NiCo_2_S_4_ in the mechanically mixed CNTs/NiCo_2_S_4_ composite. The cycleability of the CNTs/NiCo_2_S_4_ electrode was evaluated by the repeated GCD measurement at a current density of 50 A/g, as shown in Fig. 6(a). The specific capacitance of the CNTs/NiCo_2_S_4_ electrode remained stable at approximately 91.5% of the original value after 3000 charging-discharging cycles, thus showing excellent structural stability. The present work provides relativity higher specific capacitance and better rate capability even compared with the reported literatures (see Table S1).

EIS is typically used to investigate the performance of electrochemical capacitors, such as internal resistance, capacity, etc. Figure 6(b) shows the Nyquist plots of the EIS spectra of the CNTs/NiCo_2_S_4_ composite. In general, at high frequency, the intercept on the real axis represents a combined resistance (Rs) which contains the intrinsic resistance of the electrode materials, ionic resistance of the electrolyte, and the contact resistance between the electrode and current collector^26^,^31^. The present EIS plots exhibit identical Rs values of approximately 1.15 Ω and 1.32 Ω before and after 3000 cycles of the charge-discharge experiments, respectively. A quasi-semicircle was observed in the high frequency range and its diameter corresponds to the charge transfer resistance (Rct) caused by the Faradic reactions^26^,^31^. The fitted values of Rct obtained for the electrode were approximately 0.04 Ω and 0.09 Ω before and after 3000 cycles of the charge-discharge experiments, respectively. The curve of the low frequency data showed impedance of the electro-active materials, which was mostly caused by the ion diffusion within the electro-active materials^32^. Both curves showed a near vertical line in the low frequency range, which suggests low electrolyte diffusion impedance. However, a minimal slope difference was observed from the vertical diffusion lines, which demonstrates excellent capacitive performance of the electrode before and after 3000 cycles of the charge-discharge experiments. These results reveal good stability of the CNTs/NiCo_2_S_4_ composite.

According to the above results, we propose the following characteristics for the CNTs/NiCo_2_S_4_ composite: 1) CNTs work as a highly conductive support, which prevents further growth by agglomeration and ensures the full utilization of the electrode materials. Therefore, compared with pure NiCo_2_S_4_, the introduction of CNTs improved the contact between the electrode and the electrolyte; 2) the CNTs built a continuous conductive network during the hydrothermal preparation, which offered a favorable pathway for improving the conductivity and facilitating the charge transfer within the bulk electrode materials; 3) the good mechanical properties and excellent environmental stability of the CNTs’ effectively buffered the volume expansion during the charging-discharging process, thus playing an important role in the cycling stability of the electrode materials.

In summary, a novel CNTs/NiCo_2_S_4_ composite was successfully prepared via a simple chemical bath deposition combined with a post-anion exchange reaction. The CNTs built a highly conductive network that improved the conductivity and prevented further agglomeration of the hybrid composite. The synergistic effect caused by the integration of CNTs and NiCo_2_S_4_ afforded a composite with high capacitance, outstanding rate capability and excellent cycling stability as an electrode material for supercapacitors. At a current density of 1 A/g, the CNTs/NiCo_2_S_4_ composite exhibited a high capacitance of 1537 F/g, whereas at 100 A/g, the specific capacitance remained at a value of 1200 F/g. This excellent performance makes this composite a promising candidate as an electrode materials for high performance supercapacitors. Furthermore, the simple synthesis strategy presented herein can be easily extended to prepare other soft and conductive CNTs involved metal sulphide composites with controllable structure, which may also be promising electrode materials for high performance supercapacitors.

## Methods

In the experiments herein, the following materials were used: polyvinylpyrrolidone (PVP, K-30) (Aladdin Industrial Corporation), Co(NO_3_)_2_·6H_2_O, Ni(NO_3_)_2_·6H_2_O, Na_2_S·9H_2_O, urea and ammonium hydroxide (Sinopharm chemical reagent Co., Ltd.), and CNTs (Shenzhen Nanotech Port Co., Ltd.). All chemical reagents were of analytical grade and used without further purification.

The CNTs/NiCo_2_S_4_ composite was prepared according to the following steps: 1) 40 mg of CNTs were dispersed in 30 ml of PVP aqueous solution (10 mg/ml) under ultrasonic dispersion and stirring; 2) 4 mmol of Co(NO_3_)_2_·6H_2_O and 2 mmol of Ni(NO_3_)_2_·6H_2_O were dissolved in 30 ml of deionized water under stirring; 3) The above solutions were mixed together and stirred for 1 hour; 4) 0.9 g of urea and 1 ml of ammonium hydroxide were added to the above mixture; 5) After stirring for 3 hours, the precipitate was collected and washed with deionized water several times and then dried at 60 °C for 4 hours; 6) the obtained precursor was dispersed in 64 ml of 0.1 M Na_2_S aqueous solution, stirred for 10 minutes, and then the suspension was transferred into an 80 ml Teflon-lined stainless-steel autoclave; 7) The autoclave was heated at 160 °C for 12 hours and then cooled to room temperature naturally; 8) The as-obtained black precipitate was collected and washed with deionized water several times and then dried at 60 °C for 4 hours in vacuum. For comparison, pure NiCo_2_S_4_ was prepared in the same steps as above without CNTs, and the mechanically mixed CNTs/NiCo_2_S_4_ composite was also prepared.

The phase structure of the samples was characterized by using a XRD spectrometer (D8 Advanced XRD, Bruker AXS, Karsruhe, Germany) with Cu Ka radiation. The morphologies of the samples were observed by using a SEM (S-4800 Hitachi High-Technologies Corporation, Japan). Raman spectra were measured in a laser scanning confocal micro-Raman spectrometer (LabRAM HR, HORIBA, France). The surface chemical species of the samples were examined on an XPS (ESCALAB 250Xi, Thermo Fisher Scientific, USA) using Al Ka radiation of 1486.6 eV as the excitation source. The Brunauere Emmette Teller (BET) specific surface area of the samples were measured by using low-temperature sorption of nitrogen (BET, JW-BK, JWGB, China).

The electrochemical tests were performaed in 6 M KOH aqueous electrolyte solution at room temperature. The electrochemical properties of the samples were investigated using an electrochemical working station (CHI660D, Shanghai Chenhua, China). All electrochemical measurements were performaed in a three-electrode system with, the sample modified nickel foam as the working electrode (WE), platinum as the counter electrode, and saturated calomel electrode (SCE) as the reference electrode. The WE was prepared by mixing the as-prepared sample, conductive carbon black and PVDF with a mass ratio of 8:1:1. Then an appropriate amount of DMF was added and grinded for 20 min to obtain the homogeneous solution. The solution was then casted onto nickel foam (1 cm × 2 cm) to obtain the electrode. The assembled samples were pressed at 10 MPa for one minute and dried in a vacuum oven at 80 °C for 12 hours. The mass of active materials coated on each WE was approximately 1.5 mg/cm^2^.

According to the equation C = (I × Δt)/(ΔV × m), the specific capacitance (C) was calculated from the slope of each discharge curve, where I is the constant discharge current, Δt is the discharge time, ΔV is the voltage difference in discharge and m is the mass of active materials coated on each WE^33^,^34^. Electrochemical impedance spectroscopy (EIS) measurements were collected in the frequency range of 0.1–100,000 Hz by applying an AC voltage with 5 mV perturbation.

## Additional Information

**How to cite this article**: Li, D. *et al*. Facile synthesis of hybrid CNTs/NiCo_2_S_4_ composite for high performance supercapacitors. *Sci. Rep*. **6**, 29788; doi: 10.1038/srep29788 (2016).

## Supplementary Material

Supplementary Information

## Figures and Tables

**Figure 1:**
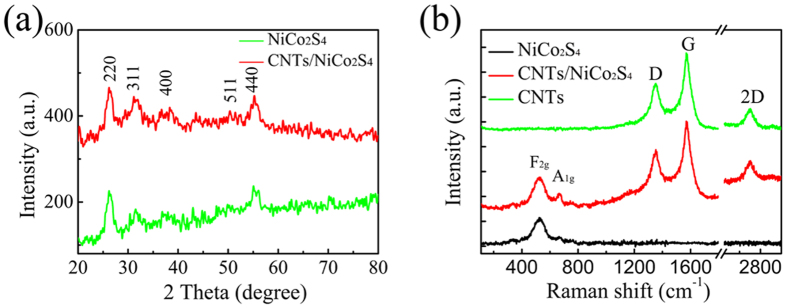
(**a**) XRD patterns and (**b**) Raman spectra of the samples.

**Figure 2:**
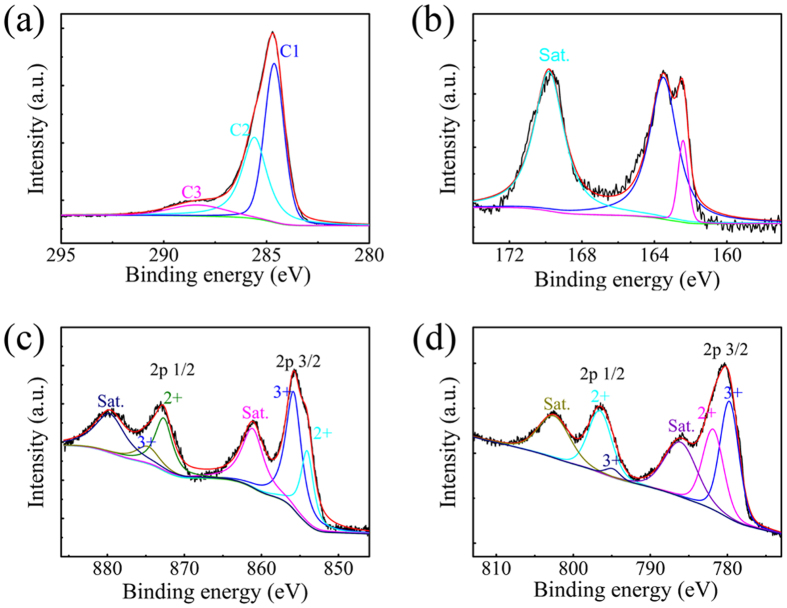
XPS spectra of the CNTs/NiCo2S4 composite: (**a**) C 1s; (**b**) S 2p; (**c**) Ni 2p; and (**d**) Co 2p.

**Figure 3:**
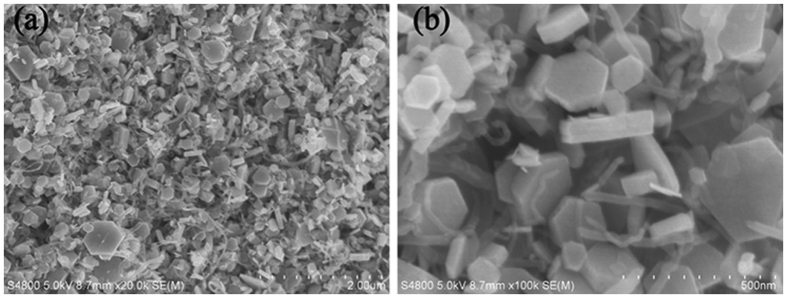
SEM morphologies of the CNTs/NiCo2S4 composite: (**a**) low magnification, (**b**) high magnification.

**Figure 4:**
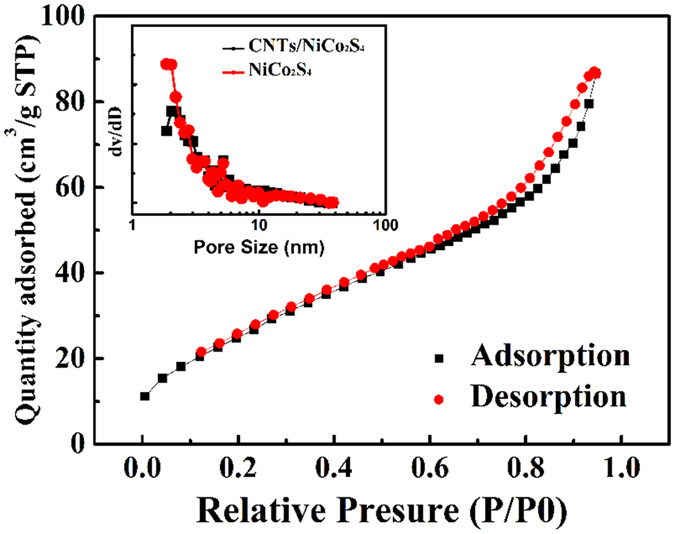
Nitrogen adsorption and desorption isotherm of the CNTs/NiCo_2_S_4_ composite. The inset shows the BJH pore size distribution curve.

**Figure 5:**
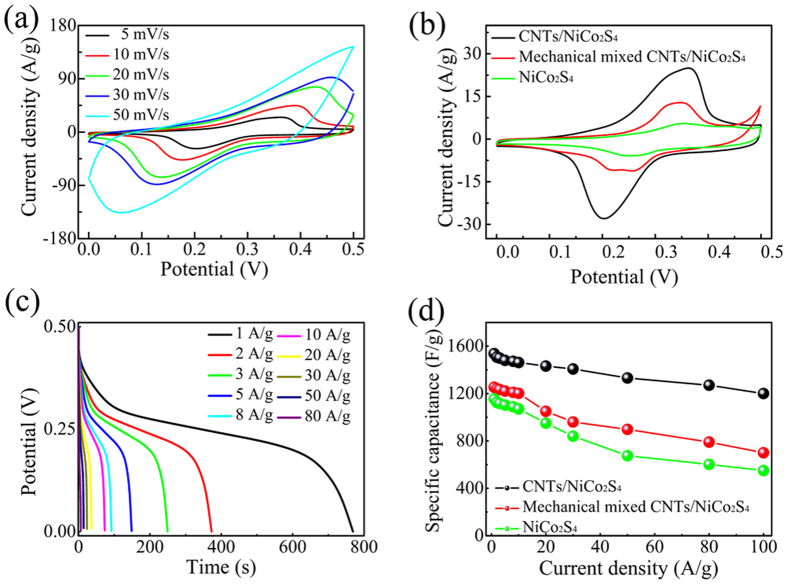
(**a**) CV curves of the CNTs/NiCo_2_S_4_ composite at different scan rate; (**b**) CV curves (at 5 mV/s) of the CNTs/NiCo_2_S_4_ composite, mechanically mixed CNTs/NiCo_2_S_4_ and pure NiCo_2_S_4_; (**c**) GCD curves of the CNTs/NiCo_2_S_4_ composite at different current densities; (**d**) specific capacitance vs. current density.

**Figure 6:**
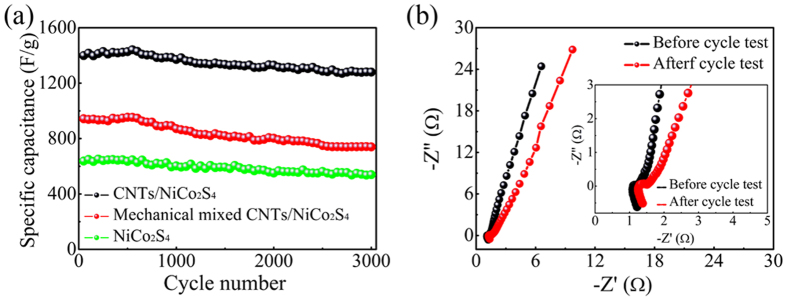
(**a**) Cycle performance of the CNTs/NiCo_2_S_4_ composite, mechanically mixed CNTs/NiCo_2_S_4_ and pure NiCo_2_S_4_ at current density of 50 A/g; (**b**) EIS plots of the CNTs/NiCo_2_S_4_ composite modified electrode in 6M KOH electrolyte before and after the cycle test.
