# Supplementary material for: Facile synthesis of hybrid CNTs/NiCo2S4 composite for high performance supercapacitors
Source: Sci Rep. 2016 Jul 11;6:29788. doi: 10.1038/srep29788 (PMC4942691; doi:10.1038/srep29788)
Supplement: Supplementary Information [file srep29788-s1.doc]

Supplementary information

**Facile synthesis of hybrid CNTs/NiCo_2_S_4_ composite for high performance supercapacitors**

Delong Li^1, 2^, Youning Gong^1, 2^, Chunxu Pan^1, 2, 3 *^

^1^ Shenzhen Research Institute, Wuhan University, Shenzhen 518057, China.

^2^ School of Physics and Technology, and MOE Key Laboratory of Artificial Micro- and Nano-structures, Wuhan University, Wuhan 430072, China

^3^ Center for Electron Microscopy, Wuhan University, Wuhan 430072, China

* Author to whom correspondence should be addressed.

E-mail: [cxpan@whu.edu.cn](mailto:cxpan@whu.edu.cn) (C. Pan); Tel: +86-027-68752481 ext. 8168

For comparison, pure NiCo_2_S_4_ was prepared in the same method without CNTs. The SEM morphology of pure NiCo_2_S_4_ was shown in Figure S1. Obviously, pure NiCo_2_S_4_ showed the same morphology as NiCo_2_S_4_ in the CNTs/NiCo_2_S_4_ composite. That was to say, the morphology of NiCo_2_S_4_ was not affected by the presence of CNTs.


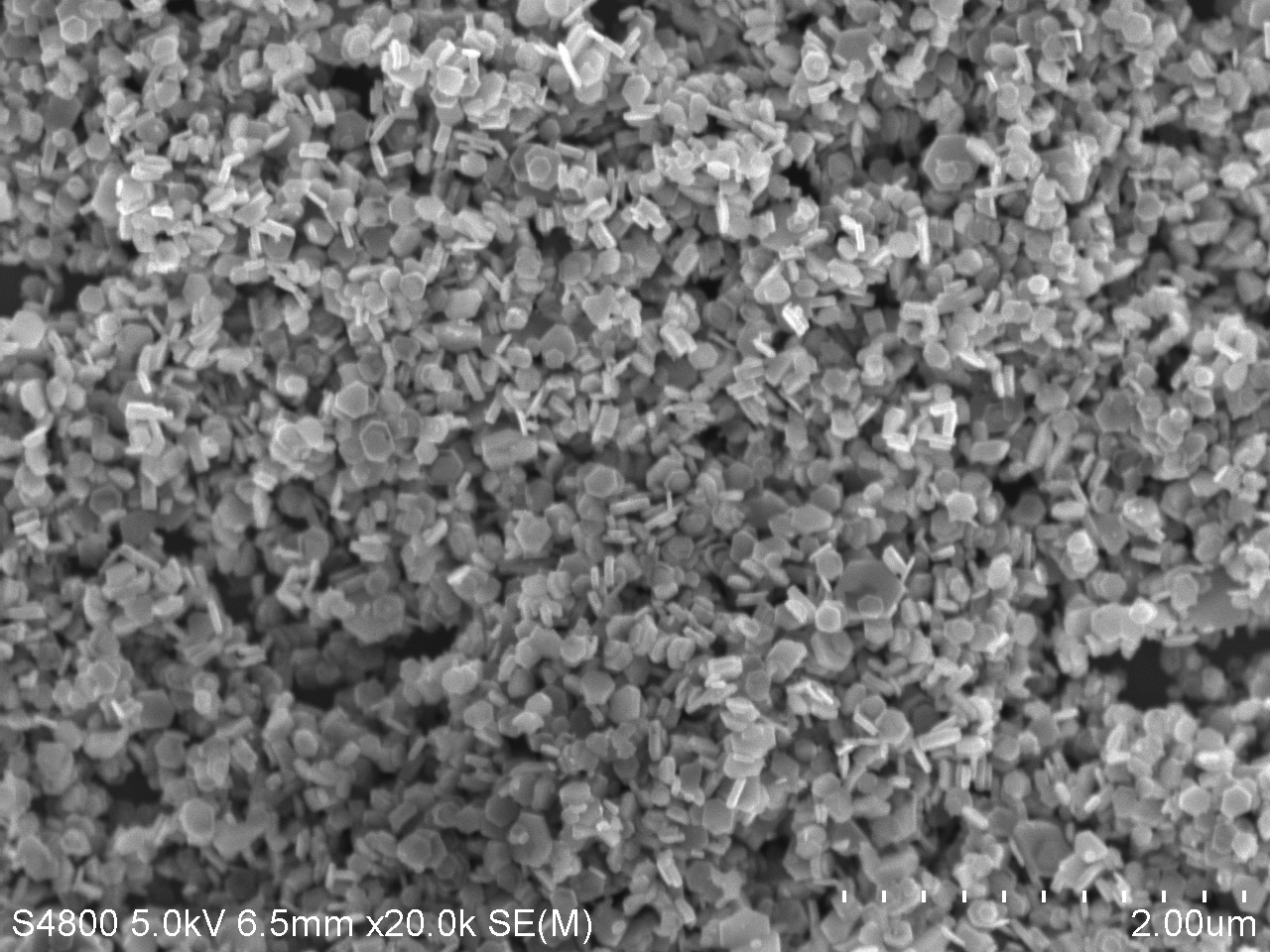

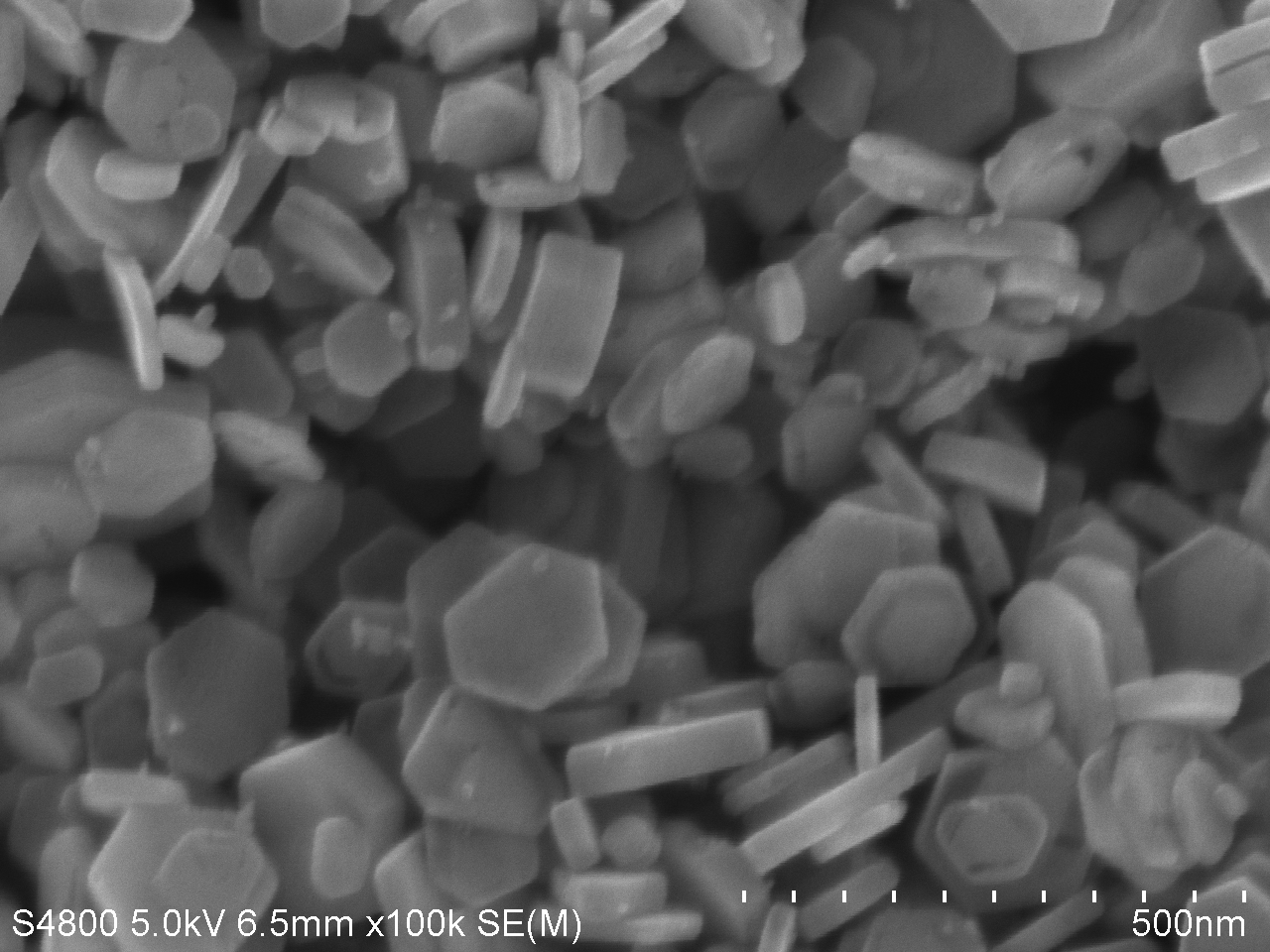


**(a)**

**(b)**

Fig. S1 SEM morphologies of NiCo_2_S_4_: (a) low magnification, (b) high magnification.

The CNTs/NiCo_2_S_4_ composite with different mass ratio of CNTs were prepared in the same experiment conditions. The samples were prepared with different mass of CNTs varied from 0 mg, 20 mg, 40 mg (presented in the main paper), and 60 mg, which were designated as NCS, CNCS-20, CNCS-40, and CNCS-60. The SEM morphology of the samples were shown in Figure S2.

Figure S3(a) illustrates the CV curves of the samples NCS, CNCS-20, CNCS-40, and CNCS-60 at a scan rate of 5 mV/s. Clearly, the enclosed area of the CNCS-40 was much larger than that of the other samples NCS, CNCS-20, and CNCS-60, which indicated that the CNCS-40 was of a lager capacitance. Figure S3(b) shows the discharge capacitance of the samples NCS, CNCS-20, CNCS-40, and CNCS-60 at a current density of 1 A/g. Similarly, the CNCS-40 delivered a higher specific capacitance than the others.


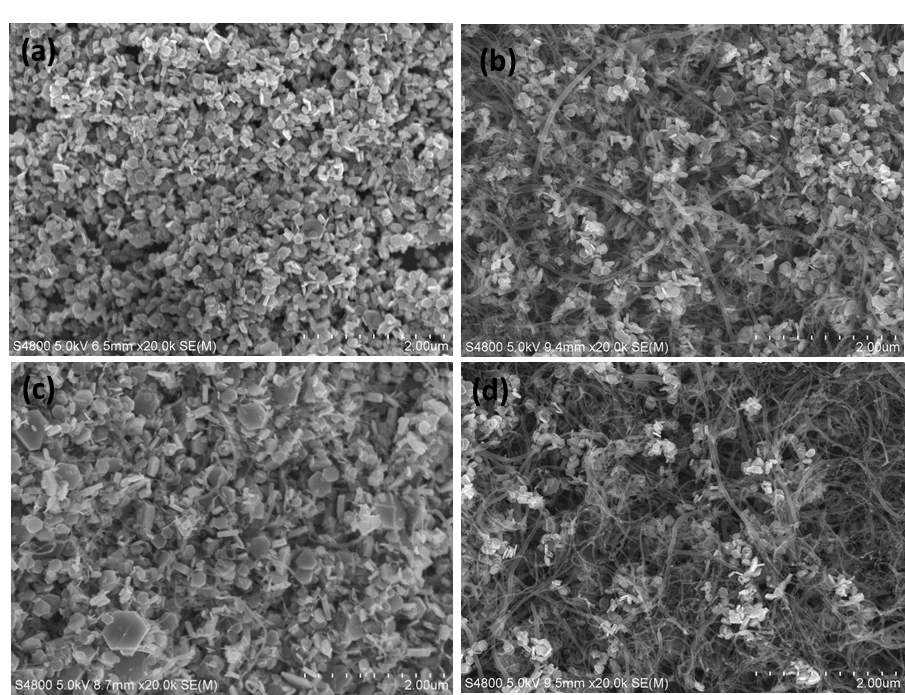


Fig. S2 SEM morphology of the CNTs/NiCo_2_S_4_ composite: (a)NCS; (b)CNCS-20; (c)CNCS-40; (d)CNCS-60







**(a)**

**(b)**

Fig. S3 (a) CV curves (at 5mV/s) and (b) GCD curves (at 1A/g) of NCS, CNCS-20, CNCS-40, and CNCS-60.

Table S1 summarized the electrochemical performance of the carbon/NiCo_2_O_4_ (or NiCo_2_S_4_) composite in the reported literatures. Comparatively, the electrochemical performance reported in this work exhibited good rate performance. In addition, the present work also provides relatively high specific capacitance and good cyclability.

Table S1 Comparison of the electrochemical performance of the carbon nano materials/NiCo_2_O_4_ (or NiCo_2_S_4_) composite with the reported results

| Material | Specific capacitance (F/g) | Rate performance | Capacity retention | Reference |
| --- | --- | --- | --- | --- |
| CNT/NiCo_2_O_4_ core/shell | 694 (1 A/g) | 82% (20 A/g) | 91% (1500 cycles) | 1 |
| NiCo_2_O_4_/graphene | 1402 (1 A/g) | 77% (20 A/g) | 76.6% (5000 cycles) | 2 |
| CNT@NiCo_2_O_4_ | 1038 (0.5 A/g) | 36% (10 A/g) | 100% (1000 cycles) | 3 |
| NiCo_2_O_4_/graphene | 1072.9 (1 A/g) | 69.2% (10 A/g) | 98.55% (3000 cycles) | 4 |
| NiCo_2_O_4_-SWCNT | 1642 (0.5 A/g) | 53.5% (20 A/g) | 94.1% (2000 cycles) | 5 |
| Carbon Nanosphere/NiCo_2_O_4_ | 1420 (1 A/g) | 72% (10 A/g) | 98.5% (3000 cycles) | 6 |
| NiCo_2_O_4_ nanoparticles on a CNT film | 828 (1 A/g) | 79% (20 A/g) | 99% (3000 cycles) | 7 |
| NiCo_2_O_4_–rGO | 870 (2 A/g) | 68.9% (20 A/g) | 90% (5000 cycles) | 8 |
| NiCo_2_O_4_/graphene | 2173 (6 A/g) | 43.9% (200 A/g) | 94% (10000 cycles) | 9 |
| NiCo_2_O_4_/CNT | 1533 (3 A/g) | 87.1% (30 A/g) | 102% (2500 cycles) | 10 |
| Carbon@NiCo_2_S_4_ | 1455 (1 A/g) | 81.1% (20 A/g) | 83% (2000 cycles) | 11 |
| NiCo_2_S_4_/NCF | 1231 (2 A/g) | 71.2% (20 A/g) | 90.4% (2000 cycles) | 12 |
| **CNT/NiCo_2_S_4_** | **1537 (1A/g)** | **78.1 % (100 A/g)** | **91.5% (3000 cycles)** | **This work** |

Reference

1. Liu W, et al. A three dimensional vertically aligned multiwall carbon nanotube/NiCo_2_O_4_ core/shell structure for novel high-performance supercapacitors. *J. Mater. Chem. A* **2,** 5100-5107 (2014).
2. Zhang C, et al. Facile preparation of flower-like NiCo_2_O_4_/three dimensional graphene foam hybrid for high performance supercapacitor electrodes. *Carbon* **89**, 328-339 (2015).
3. Cai F, et al. Hierarchical CNT@ NiCo_2_O_4_ core–shell hybrid nanostructure for high-performance supercapacitors. *J. Mater. Chem. A* **2**, 11509-11515 (2014).
4. Xu J, et al. Facile preparation of NiCo_2_O_4_ nanobelt/graphene composite for electrochemical capacitor application. *Electrochimica Acta* **166**, 206-214 (2015).
5. Wang X, et al. Nickel cobalt oxide-single wall carbon nanotube composite material for superior cycling stability and high-performance supercapacitor application. *J. Phys. Chem. C* 2012, **116**, 12448-12454.
6. Li D, et al. Facile Synthesis of Carbon Nanosphere/NiCo_2_O_4_ Core-shell Sub-microspheres for High Performance Supercapacitor. *Sci. rep.* **5**, 12903 (2015).
7. Xu S, et al. Fabrication of NiCo_2_O_4_ and carbon nanotube nanocomposite films as a high-performance flexible electrode of supercapacitors. *RSC Adv.* **5**, 74032-74039 (2015).
8. Umeshbabu E, et al. Synthesis of mesoporous NiCo_2_O_4_–rGO by a solvothermal method for charge storage applications. *RSC Adv*. **5**, 66657-66666 (2015).
9. Zhou J, et al. Two-dimensional NiCo_2_O_4_ nanosheet-coated three-dimensional graphene networks for high-rate, long-cycle-life supercapacitors. *Nanoscale* **7**, 7035-7039 (2015).
10. Li X, et al. Three-dimensional hierarchical self-supported NiCo_2_O_4_/carbon nanotube core–shell networks as high performance supercapacitor electrodes[J]. *RSC Adv.* **5**, 7976-7985 (2015).
11. Li L, et al. Carbon@NiCo_2_S_4_ nanorods: an excellent electrode material for supercapacitors, *RSC Adv.* **5**, 83408-83414 (2015).
12. Shen L, et al. NiCo_2_S_4_ Nanosheets Grown on Nitrogen-Doped Carbon Foams as an Advanced Electrode for Supercapacitors, *Adv. Energy Mater.* **5**, 1400977 (2015).
